# Characterization of Banana *SNARE* Genes and Their Expression Analysis under Temperature Stress and Mutualistic and Pathogenic Fungal Colonization

**DOI:** 10.3390/plants12081599

**Published:** 2023-04-10

**Authors:** Bin Wang, Yanbing Xu, Shiyao Xu, Huan Wu, Pengyan Qu, Zheng Tong, Peitao Lü, Chunzhen Cheng

**Affiliations:** 1 College of Horticulture, Fujian Agriculture and Forestry University, Fuzhou 350002, China; 2 College of Horticulture, Shanxi Agricultural University, Jinzhong 030801, China; 3 Institute of Tropical Bioscience and Biotechnology, Chinese Academy of Tropical Agricultural Sciences, Haikou 571101, China

**Keywords:** banana, SNARE, temperature stress, *Serendipita indica*, *Fusarium oxysporum* f. sp. *cubense*

## Abstract

SNAREs (soluble N-ethylmaleimide-sensitive-factor attachment protein receptors) are engines for almost all of the membrane fusion and exocytosis events in organism cells. In this study, we identified 84 *SNARE* genes from banana (*Musa acuminata*). Gene expression analysis revealed that the expression of *MaSNARE*s varied a lot in different banana organs. By analyzing their expression patterns under low temperature (4 °C), high temperature (45 °C), mutualistic fungus (*Serendipita indica*, Si) and fungal pathogen (*Fusarium oxysporum* f. sp. *Cubense* Tropical Race 4, *Foc*TR4) treatments, many *MaSNAREs* were found to be stress responsive. For example, *MaBET1d* was up-regulate by both low and high temperature stresses; *MaNPSN11a* was up-regulated by low temperature but down-regulated by high temperature; and *Foc*TR4 treatment up-regulated the expression of *MaSYP121* but down-regulated *MaVAMP72a* and *MaSNAP33a*. Notably, the upregulation or downregulation effects of *Foc*TR4 on the expression of some *MaSNAREs* could be alleviated by priorly colonized Si, suggesting that they play roles in the Si-enhanced banana wilt resistance. *Foc* resistance assays were performed in tobacco leaves transiently overexpressing *MaSYP121*, *MaVAMP72a* and *MaSNAP33a*. Results showed that transient overexpression of *MaSYP121* and *MaSNPA33a* suppressed the penetration and spread of both *Foc*1 (*Foc* Race 1) and *Foc*TR4 in tobacco leaves, suggesting that they play positive roles in resisting *Foc* infection. However, the transient overexpression of *MaVAMP72a* facilitated *Foc* infection. Our study can provide a basis for understanding the roles of *MaSNAREs* in the banana responses to temperature stress and mutualistic and pathogenic fungal colonization.

## 1. Introduction

Vesicle trafficking, a conserved intracellular transport mechanism in eukaryotic cells, is crucial for the plant growth and development and plant adaptations to various external and internal stresses [1,2]. It mainly involves four steps, i.e., budding, transporting, tethering and the fusion of vesicles with acceptor membranes [3]. Soluble N-ethylmaleimide-sensitive-factor attachment protein receptors (SNAREs) participate and function in almost all of the membrane fusion processes in cells [4,5,6,7]. According to their localization during membrane fusion, SNAREs can be divided into vesicle-SNARE (v-SNAREs) and target-SNARE (t-SNAREs) types [8]. However, since some SNARE protein displayed variable localizations, somewhile this classification would be inaccurate [9]. SNARE proteins contain conserved SNARE domains composed of about 60 amino acids. According to whether an arginine (R) or a glutamine (Q) exists in the core sequence of this domain, SNAREs can be classified into R-SNAREs and Q-SNAREs [10]. Moreover, the Q-SNAREs can be further classified into Qa-, Qb-, Qc- and Qbc-SNAREs [11].

Since the first discovery of VAMP-1 (synaptic vesicle-associated membrane protein 1; a neuron-specific synaptic vesicle-associated internal membrane protein) in the electric organ of *Torpedo* in 1988 [12], many core SNAREs and their homologous proteins, such as SNAP-25 [13] and Syntaxin [14], have been successively identified. Generally, SNAREs exist as a large and dispersed multigene family with more than 20 members per genome. For example, there are 38, 26 and 25 *SNARE* genes in the genome of *Homo sapiens* [15], *Drosophila melanogaster* [16] and *Saccharomyces cerevisiae* [17], respectively. Due to the evolution and increasement of multicellularity, the numbers of *SNARE* members in green plant genomes became more abundant [18]. In the model plant, *Arabidopsis thaliana*, 64 *SNARE* members are identified. Rice (*Oryza sativa* L.), black cottonwood (*Populus trichocarpa*), Tomato (*Solanum lycopersicum* L.) and wheat (*Triticum aestivum* L.) have 60 [19], 69 [20], 63 [21] and 173 [22] *SNARE* members, respectively.

SNAREs display miscellaneous functions in regulating substance transport, participating in cytoplasmic division and mediating ion channel stability [23]. The Arabidopsis *SYP111* deletion mutants displayed abnormal vesicle trafficking and inhibited cell wall formation and cytokinesis [24]. Arabidopsis VAMP721 and VAMP722 are involved in the vesicular trafficking between the plasma membrane and the *cis*/*trans*-Golgi network (CGN/TGN), playing roles in facilitating the formation of cell plates and the transportations of proteins and many other molecules during cell division [25,26]. VAMP721 can interact with and inhibit potassium channel-related proteins (such as KAT1 and KC1) and thereby influence potassium ion transport [27].

SNAREs play important roles in plant stress responses. It has been reported that SNARE proteins function in regulating the Ca^2+^ signaling pathway and indirectly influence the regulation of ABA signaling on K^+^ and Ca^2+^ channels to enhance the salt, alkali and drought resistances of plants [28]. Recently, their participations in the plant immunity responses to pathogens, especially fungal pathogens, have been reported. The accumulation of pathogenesis-related protein PR1 in *SYP132*-silenced transgenic tobacco plants was significantly suppressed [29]. The SNARE complex, consisting of PEN1 (SYP121), SNAP33 and VAMP721/VAMP722, is reported to be indispensable for the defense responses against powdery mildew fungi infection in Arabidopsis [7]. This complex can assist cell exocytosis in the powdery mildew infection sites, and thereby resist the invasion of pathogens [30]. Positive functions of Barley (*Hordeum vulgare* L.) HvROR2 [31] and tomato (*Solanum lycopersicum* L.) SlPEN1 [32] in the plant-powdery mildew interactions have also been confirmed. In addition, AtSEC11 was reported to have the ability of improving the SNARE complex stability by competing with VAMP721 and SNAP33 for PEN1 binding, thereby improving the powdery mildew resistance of Arabidopsis [33].

Banana (*Musa* spp.) is one of the most important fruit trees in the world. As a tropical fruit tree, it is very sensitive to high and low temperature stresses [34]. Moreover, the banana industry has been severely threatened by the devastating Fusarium wilt (FW) disease caused by soil-borne fungus *Fusarium oxysporum* f. sp. *cubense* (*Foc*) [35,36]. Previously, we found that *Serendipita indica* (Si), a culturable arbuscular mycorrhizal fungus (AMF)-like endophytic fungus, could promote banana plant growth and improve its resistance to both temperature stress and FW disease [37,38,39]. Although SNARE proteins have been extensively reported to play roles in plant stress responses, till now, there are still few reports on banana SNAREs. In this study, to explore the possible contributions of SNAREs to temperature stress and mutualistic and pathogenic fungal colonization, genome-wide identification and characterization of banana *SNARE* genes was performed, and their expression patterns in different organs, in leaves treated with low temperature and high temperature, and in roots treated with *S. indica* and *Foc* tropical race 4 (*Foc*TR4), were investigated. Furthermore, the effects of three roots that were highly expressed and *S. indica*- and *Foc*TR4-responsive *MaSNAREs* (*MaSYP121*, *MaVAMP72a* and *MaSNAP33a*) on the infection of *Foc*1 and *Foc*TR4 were studied using the tobacco leaf transient expression method. The results obtained in this study will be helpful in clarifying the roles of MaSNAREs in banana stress responses.

## 2. Results

### 2.1. Physicochemical Properties of the Identified MaSNAREs

In total, 84 *SNARE* genes were identified from the *M. acuminata* genome (Supplemental Table S1). They were named according to their closest Arabidopsis homologous genes. For the banana *SNARE* genes that shared the same homologous gene, they were orderly named based on their chromosome location information. For example, the homologous genes of Arabidopsis *SYP121*, *VAMP721* and *SNAP33* in banana were named as *MaSYP121*, *MaVAMP72a-c* and *MaSNAP33a-e*, respectively.

The CDS lengths of *MaSNAREs* ranged from 159 bp to 3336 bp, and their encoded proteins contained 52 aa~1111 aa, with a molecular weight of 6057.79 kDa~121,494.46 kDa and the isoelectric point (PI) of 4.71~9.93. Their protein instability coefficient ranged from 29.68 to 76.84 and their lipid solubility index ranged from 60.87 to 123.48, respectively. Among them, MaSYP125a, MaVTI1b, MaNPSN13b, MaNPSN13c, MaBET1b and MaVAMP71b were hydrophobic proteins, and the others were hydrophilic proteins.

### 2.2. Subcellular Localization Prediction of MaSNAREs

Subcellular localization prediction results showed that there were 30, 14, 13, 13, 8 and 5 MaSNAREs localized in the plasma membrane, vacuole, endoplasmic reticulum, Golgi apparatus, nucleus and cytoplasmic matrix (Table S1), respectively. In consistence with the Arabidopsis SNAREs [1], the plasma membrane-localized MaSNAREs take the largest part.

### 2.3. Phylogenetic Analysis of MaSNARE Proteins

**Figure 1:**
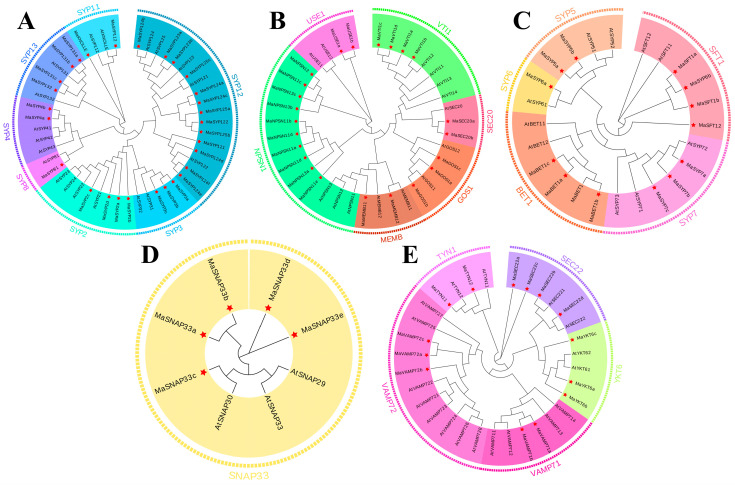
Phylogenetic trees constructed using Qa-SNARE (**A**), Qb-SNARE (**B**), Qc-SNARE (**C**), Qbc-SNARE (**D**), and R-SNARE (**E**) subfamily members from *Musa acuminata* (Ma) and *Arabidopsis thaliana* (At).

By using the 84 MaSNAREs and the 64 Arabidopsis SNAREs sequences, a phylogenetic tree was constructed. Results showed that MaSNAREs could be classified into five subfamilies, including Qa (containing 29 members), Qb (22 members), Qc (14 members), Qbc (5 members) and R (14 members) subfamilies (Figure 1 and Supplemental Figure S1). The banana Qa subfamily could be further divided into seven groups (SYP11, SYP12, SYP13, SYP2, SYP3, SYP4 and SYP81, with 2, 13, 4, 4, 3, 2 and 1 MaSNARE members, respectively). The number of the banana SYP12 members is much larger than that of the Arabidopsis SYP12 (5). The banana Qb subfamily could be divided into GOS1, SEC20, MEMB, USE1, VTI1 and NPSN1 groups, containing 3, 2, 2, 2, 4 and 9 members, respectively. The banana Qc subfamily could be divided into SFT1, BET1, SYP5, SYP6 and SYP7 groups, containing 3, 4, 2, 2 and 3 members, respectively. The banana Qbc subfamily contained a single SNAP33 group with five MaSNARE members. And the banana R subfamily could be divided into TYN1, VAMP71, VAMP72, YKT6 and SEC22 groups, containing 2, 2, 3, 3 and 4 members, respectively.

### 2.4. Conserved Motifs in MaSNAREs and Gene Structures of Their Encoding Genes

**Figure 2:**
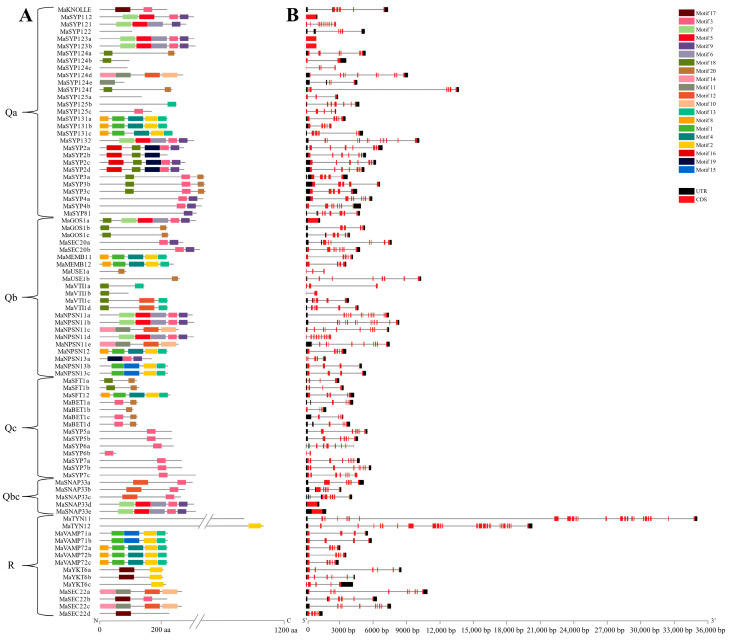
Conserved motifs (**A**) in MaSNAREs and gene structures of their encoding genes (**B**). Qa, Qb, Qc, Qbc and R represent five subfamilies of banana SNAREs.

A total of 20 conserved motifs were identified from MaSNARE proteins (Figure 2A). Among the Qa subfamily MaSNAREs, all SYP13 group members contained Motif8, 1, 4, 2 and 13; all SYP2 group members contained Motif16, 18 and 19; all SYP3 group members contained Motif18 and Motif3; and all SYP4 group members contained Motif3 and Motif9. Among the Qb subfamily members, all GOS1 and VTI1 group members contained Motif18; SEC20 and SYP4 group members contained the same motifs; MEMB11, MEMB12 and SYP13 group members contained the same motifs in the same order; USE1 group members contained only Motif20; of the NPSN11 group members, MaNPSN11a, b and d had four motifs ordering in Motif7-5-6-3-9, while MaNPSN11c and MaNPSN11e had four other motifs in the order of Motif14-11-12-10; of the MaNPSN13 group members, MaNPSN13a had three motifs ordering in Motif19-3-9, while MaNPSN13b and MaNPSN13c had four motifs ordering in Motif1-15-2-13. Among the Qc subfamily MaSNAREs, Motif18 and Motif20 were found in SFT1 group members, and all BET1, SYP5 and SYP7 group members contained Motif3. In the Qbc subfamily MaSNAREs, there were two types of motif orders for SNAP33 group, i.e., Moitf12-3 and Motif7-5-6-3-9. In the R subfamily, the motif ordering of VAMP71 group members was Motif1-15-2-13 and that of VAMP72 group members was Motif5-1-4-2-13. In addition, no conserved motif was identified in MaSYP122, MaSYP124c, MaSYP125a and MaTYN11.

Gene structure analysis results showed that the exon numbers of Qa subfamily *MaSNAREs* ranged from 1 to 12, with *MaSYP121* having the most exons (12), and *MaSYP123a* and *MaSYP123b* having a single exon (Figure 2B). *MaSYP131a-c* all contained five exons, *MaSYP2a-d* contained seven exons, *MaSYP3a-c* contained five exons, and *MaSYP4a-b* contained eight exons. The exon numbers of Qb subfamily *MaSNAREs* ranged from 1 to 13. *MaNPSN11a*, *MaNPSN11b* and *MaNPSN11d* had the most exons (13), and *MaGOS1a* had one exon. The exon numbers of Qc subfamily *MaSNAREs* ranged from 2 to 9, *MaSYP7a*, *MaSYP7b* and *MaSYP7c* had the most exons (9), and *MaSYP6b* had the least exons (2). The exon numbers of Qbc subfamily *MaSNAREs* ranged from 1 to 6. *MaSNAP33a-e* had 5, 5, 6, 1, and 1 exons, respectively. The exon numbers of R subfamily *MaSNAREs* ranged from 5 to 24, with *MaTYN11* and *MaTYN12* having the most exons (24), and *MaYKT6c* and *MaSEC22d* having the least exons (5).

### 2.5. Promotor Cis-Acting Elements Analysis Results of MaSNAREs

**Figure 3:**
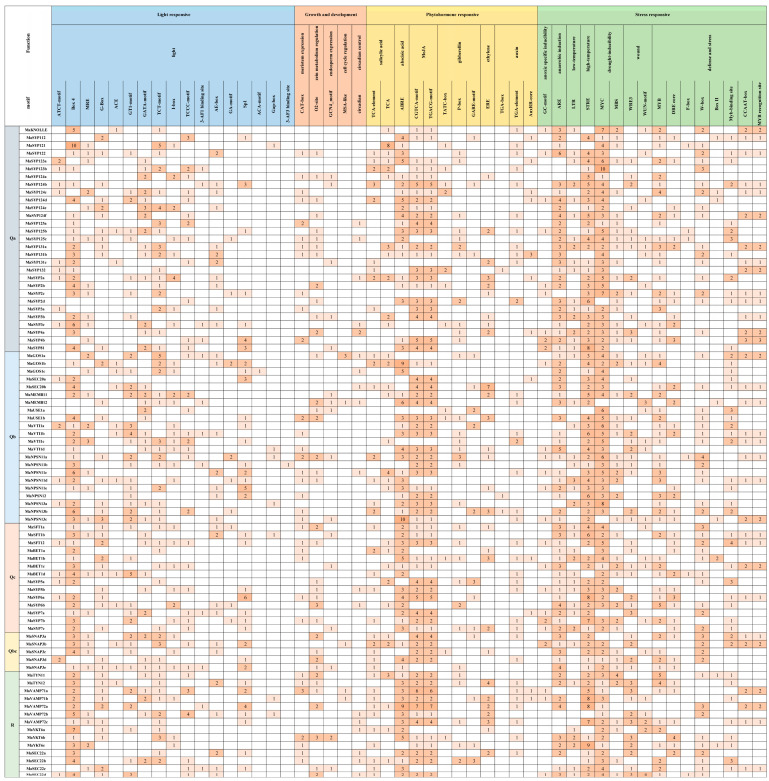
Predicted *cis*-acting elements in the promoters of *MaSNARE* genes. Qa, Qb, Qc, Qbc and R represent five SNARE subfamilies of banana. *Cis*-acting elements related to unknown functions were not shown.

A large number of light-, phytohormone-, and stress-responsive, and growth and development-related *cis*-acting elements were identified in the promoters of *MaSNAREs* (Figure 3). Moreover, there were also many *cis*-acting elements related to some unknown functions (Supplemental Table S2).

In total, we identified 17 types of light-responsive elements in the promoters of *MaSNAREs*. Notably, 68 (80.95%) *MaSNAREs* contained the light-responsive Box4 elements in their promoters, and the *MaSYP121* promoter contained the largest number of Box4 element (in total of 10). In addition, 50 (59.52%) *MaSNARE* promoters contained CTT-motif elements.

Five types of growth and development-related elements (CAT-box, O2 (Opaque2)-site, GCN4_motif, MSA-like and circadian) were identified in the promoters of *MaSNAREs*. Among them, zein metabolism regulation O2-site element was found in the promoters of 37 (44.05%) *MaSNAREs*; the meristem expression-related elements CAT-box was identified in the promoters of 33 (39.29%) *MaSNAREs*; the circadian clock regulation-related element circadian was found in the promoters of 18 (21.43%) *MaSNAREs*; the endosperm expression-related element GCN4_motif was found in the promoters of 16 (19.05%) *MaSNAREs*; and the cell cycle regulation-related MSA-like motif was found in the promoters of 5 (5.95%) *MaSNAREs*.

The *MaSNAREs* promoters contained *cis*-acting elements related to the responses to six kinds of phytohormones, including gibberellin (GA), methyl jasmonate (MeJA), salicylic acid (SA), auxin, abscisic acid (ABA) and ethylene (ET). There were three kinds of GA responsive elements (P-box, TATC-box and GARE-motif), two kinds of MeJA responsive elements (TGACG-motif and CGTCA-motif), two kinds of SA responsive elements (TCA-element and TCA), three kinds of auxin responsive elements (TGA-box, TGA-element and AuxRR-core), one kind of ABA responsive element (ABRE) and one kind of ethylene responsive element (ERE) in the *MaSNARE* promoters. Interestingly, the promoters of *MaNPSN13b*, *MaVAMP71b* and *MaVAMP72a* contained elements related to all the six phytohormones. Moreover, there were 66 (78.57%), 65 (77.38%) and 50 (59.52%) *MaSNARE* contained MeJA responsive TGACG-motif and CGTCA-motif elements, ABA responsive elements and SA responsive elements in their promoters, respectively. It was noted that *MaSYP121* contained eight SA-responsive TCA elements. In addition, there were 44 (52.38%), 36 (42.86%) and 28 (33.33%) *MaSNAREs* contained auxin-, GA-, and ET-responsive elements in their promoters, respectively.

The promoters of *MaSNAREs* also contained seven types of stress-responsive elements, including anoxic specific inducibility-, anaerobic induction-, low temperature-, high temperature-, drought inducibility-, wound-, defense- and stress-related elements. Notably, the promoters of *MaSYP122*, *MaSYP4b*, *MaNPSN11a* and *MaSEC22d* contained all the seven types of stress-responsive elements. Except for *MaSYP2d*, *MaMEMB12*, *MaNPSN13c*, *MaSYP7a* and *MaSNAP33a*, all the other 79 (94.05%) *MaSNAREs* contained drought inducibility-related MYC element in their promoters. High-temperature inducibility-related STRE element was found in the promoters of 76 (90.48%) *MaSNAREs*. Anaerobic induction-related ARE element was predicted in the promoters of 71 (84.52%) *MaSNAREs*. Additionally, there were 58 (69.05%), 52 (61.90%) and 41 (48.81%) *MaSNAREs* contained MYB, W-box and LTR element in their promoters, respectively.

### 2.6. Chromosome Localization and Collinearity Analysis Results for MaSNAREs

**Figure 4:**
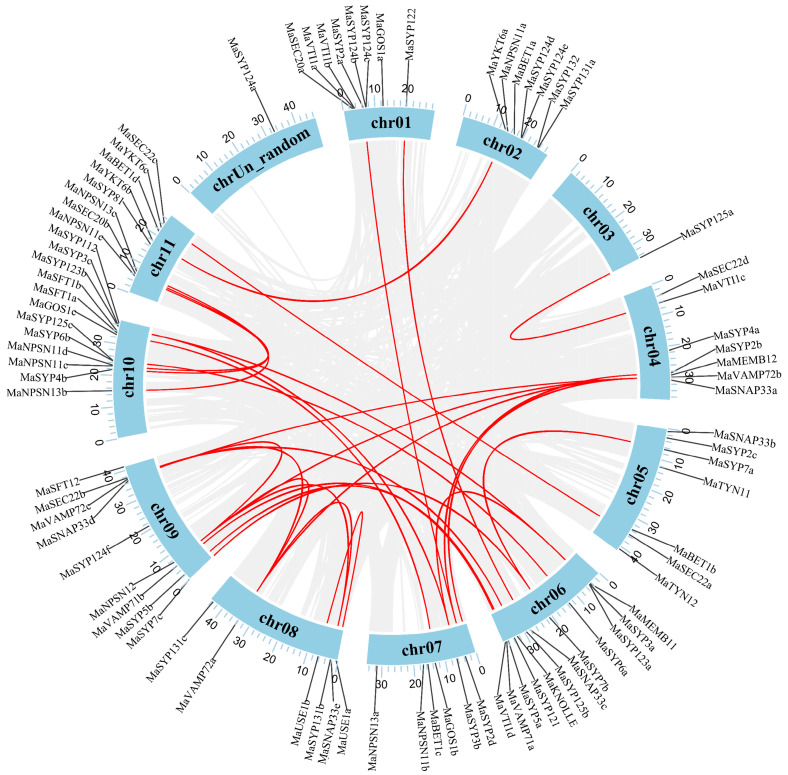
Chromosome localization and collinear distribution analysis results of *MaSNAREs*. Chr: chromosome; Ma: *Musa acuminata*. The gray lines represent segmental duplicated gene pairs in the whole banana genome; the red lines represent segmental duplicated *MaSNAREs* gene pairs.

Chromosome localization analysis results showed that *MaSNAREs* were unevenly distributed on all of the 11 chromosomes of *M. acuminata* (Figure 4). The accounts of *MaSNARE* members on Chr6 and Chr10 were the largest (12 members for each chromosome). Chr9 had nine members; Chr1 and 11 both had eight members; Chr2, 4 and 5 had seven members; Chr7 and 8 both had six members; and Chr3 had one member. In addition, *MaSYP124a* was found to be localized on chrUn_random. The accounts of Qa subfamily *MaSNAREs* on Chr6 and Chr10 were both the largest (five members for each chromosome). Qb subfamily *MaSNAREs* were distributed on all the chromosomes except Chr3 and Chr5. Qc subfamily *MaSNAREs* were unevenly distributed on Chr2, 5, 6, 7, 9, 10 and 11. Qbc subfamily *MaSNAREs* had one member each on Chr4, 5, 6, 8 and 9. And the R subfamily *MaSNAREs* were distributed on Chr2, 4, 5, 6, 8, 9, and 11.

By using MCScanX software, the gene duplication events that occurred in the *MaSNAREs* were analyzed. A total of 23 segmental duplication gene pairs involving in 34 *MaSNAREs* were identified, but no tandem duplication gene pair was found. Of the 23 segmental duplicated gene pairs, six gene pairs were from the R subfamily (*MaSEC22a*, *22c*; *MaVAMP71a*, *71b*; *MaVAMP72a*, *72c*; *MaVAMP72b*, *72a*; *MaYKT6a*, *6b*; *MaVAMP72b*, *72c*), five gene pairs were from the Qa subfamily (*MaSYP123a*, *123b*; *MaSYP2a*, *2d*; *MaSYP3a*, *3b*; *MaSYP3a*, *3c*; *MaSYP3b*, *3c*), three gene pairs were from the Qb subfamily (*MaNPSN11c*, *11e*; *MaNPSN13b*, *13c*; *MaUSE1a*, *1b*), three gene pairs were from the Qc subfamily (*MaSYP5a*, *5b*; *MaSYP7a*, *7b*; *MaSYP7b*, *7c*), and the remaining six gene pairs were from different subfamilies (*MaKNOLLE*/*MaSEC22b*, *MaNPSN12*/*MaVAMP72c*, *MaSYP125a*/*MaVTI1c*, *MaSYP4b*/*MaSEC20b*, *MaVAMP72a*/*MaNPSN12* and *MaVAMP72b*/*MaNPSN12*). No duplicated gene pair was identified from the Qbc subfamily. Notably, two duplicated gene pairs were found to be involving three *MaSNARE* members (*MaSYP3a*, *3b* and *3c*; *MaVAMP72b*, *72a* and *72c*).

The *Ka*/*Ks* values of these gene pairs ranged from 0.0235 to 0.4168 (Table S3), indicating that the evolution of this banana gene family was mainly influenced by strong purification selection pressure. The *Ka* and *Ks* values of the duplicated gene pairs were further used to calculate their divergence times. It was revealed that these gene duplication events occurred at 55.27~115.62 Mya (Table S3).

### 2.7. Gene Expression Analysis Results

#### 2.7.1. Expression Analysis Results of MaSNAREs in Four Different Organs

**Figure 5:**
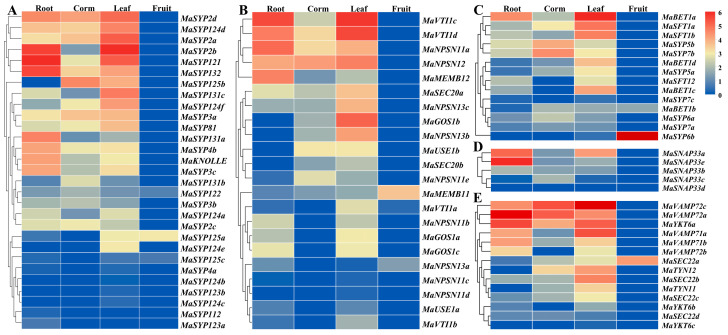
Expression heatmap for *MaSNARE* genes in different banana organs. (**A**–**E**) Qa, Qb, Qc, Qbc and R subfamily, respectively. For the heatmap drawing, the average FPKM values of three biological replications were used. The redder the color, the higher the expression level; the bluer, the lower the expression level.

Based on our transcriptome data, the spatial expression variations of *MaSNAREs* in banana root, corm, leaf and fruit were investigated (Figure 5). There were 69, 58, 81 and 9 members expressed in root, corm, leaf and fruit, respectively. *MaSEC22a*, *MaBET1b*, *MaMEMB11* and *MaSYP122* expressed in all the four banana organs, while *MaSNAP33d* showed no expression in any of these organs. The expression level of *MaVAMP72a* in root was the highest among all of the *MaSNAREs*, followed by *MaSNAP33e* and *MaSYP121*. The expression level of *MaVAMP72c* was the highest in corm among all of the *MaSNAREs*, followed by *MaVAMP72a*. The expression level of *MaVAMP72c* was the highest in leaf among all of the *MaSNAREs*. And the expression level of *MaSYP6b* ranked the first in fruit.

The expression levels of different group members from the same subfamily in different organs also varied a lot. In the Qa subfamily, the expression level of *MaSYP121* in root was the highest, followed by *MaSYP2b*. The expression level of *MaSYP2b* in leaf was the highest, followed by *MaSYP121*. The expression of *MaSYP125b* and *MaSYP125a* was the highest in corm and fruit, respectively. In the Qb subfamily, the expression of *MaVTI1c* was the highest in both root and leaf; the expression level of *MaNPSN12* was the highest in corm, followed by *MaVTI1d*; the expression of *MaMEMB11* was the highest in fruit. In the Qc subfamily, *MaBET1a* showed the highest expression in root among all the Qc members, followed by *MaSYP5b*; the expression level of *MaBET1a* was the highest in leaf, followed by *MaSFT1a*; the expression level of *MaSYP7b* in corm was the highest, followed by *MaSYP5b*; the expression level of *MaSYP6b* was the highest in fruit. In the Qbc subfamily, *MaSNAP33e* expressed the highest in both root and corm, followed by *MaSNAP33a*; the expression level of *MaSNAP33a* was the highest in leaf; but none of the five members expressed in fruit. In the R subfamily, *MaVAMP72a* expressed the highest in roots; the expression of *MaVAMP72c* was the highest in both corm and leaf; and *MaSEC22a* was the only R subfamily member that expressed in fruit.

#### 2.7.2. Expression Analysis of MaSNAREs in Banana Leaves under Low and High Temperature Treatments

**Figure 6:**
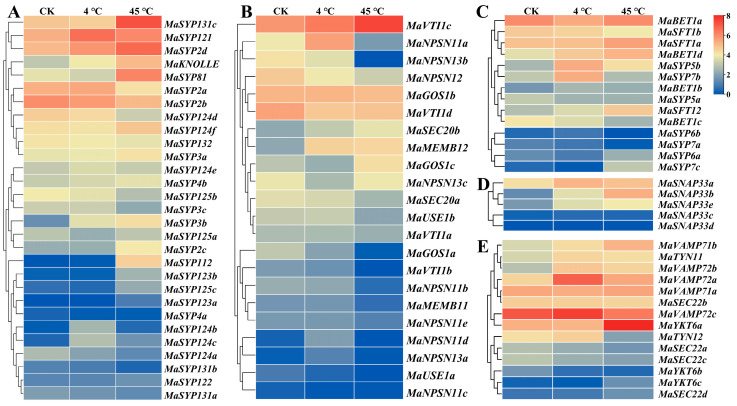
Expression heatmap for the expression of *MaSNARE* genes in leaves under low (4 °C) and high temperature (45 °C) treatments. (**A**–**E**) Qa, Qb, Qc, Qbc and R subfamily, respectively. CK: leaves from plants grown at 28 °C. Average FPKM values of three biological replications were used for the heatmap drawing. The redder the color, the higher the expression level; the bluer, the lower the expression level.

We further studied the influences of low and high temperature treatments on the expression of *MaSNAREs* in banana leaves (Figure 6). The expression levels of *MaVAMP72a*, *MaNPSN11a*, *MaSYP121* and *MaBET1d* increased more than twofold by low temperature treatment, accounting for 5.62-, 3.33-, 2.58-, and 2.1-fold of CK, respectively. Among them, only the *MaSYP121* promoter did not contain a low-temperature responsive element LTR. The expression of *MaNPSN13c* and *MaNPSN13b* in low temperature treated leaves, however, significantly decreased to 39.74% and 41.52% of CK, respectively. The expression levels of *MaYKT6a*, *MaSYP81*, *MaSYP131c*, *MaSYP2d* and *MaBET1d* increased by high temperature treatment, accounting for 7.05-, 5.74-, 5.63-, 3.38- and 2.92-fold of CK, respectively. Among them, only the *MaBET1d* promoter did not have a high-temperature responsive element STRE. The expression of *MaTYN12*, *MaNPSN11a* and *MaNPSN12* in high temperature treated leaf decreased to 16.1%, 17.25% and 33.64% of CK, respectively. Interestingly, the expression of *MaNPSN11a* was up-regulated by low temperature but down-regulated by high temperature.

Obvious expression change pattern differences were found among different subfamily members and members from the same subfamily under temperature stress. In the Qa subfamily, *MaSYP121* expressed the highest in low temperature treated leaf, followed by *MaSYP2d* and *MaSYP2a*; the expression level of *MaSYP131c* was the highest in the high temperature treated leaf, followed by *MaSYP121* and *MaSYP81*; the expression level of *MaSYP121* in low temperature and high temperature treated banana leaf accounted for 2.58- and 1.78-fold of CK, respectively; the expression of *MaSYP2d* in low temperature and high temperature treated banana leaf accounted for 1.77- and 3.38-fold of CK, respectively. In the Qb subfamily, the expression level of *MaVTI1c* was the highest in both high and low temperature treated leaves among all the Qb subfamily members; its expression in high temperature treated leaf was about 3.15-fold of CK. In the Qc subfamily, the expression of *MaBET1a* was the highest in both high and low temperature treated leaves, followed by *MaSFT1a*; the expression level of *MaSFT1a* in low temperature treated banana leaf decreased to 64.62% of CK. In the Qbc subfamily, the expression of *MaSNAP33a* was the highest in low temperature treated leaves, followed by *MaSNAP33b* and *MaSNAP33e*; the expression level of *MaSNAP33b* in high temperature treated leaves was the highest among the Qbc members, followed by *MaSNAP33a* and *MaSNAP33e*; the expression levels of *MaSNAP33a*, *MaNAP33b*, *MaNAP33c* and *MaSNAP33e* were up-regulated by both high and low temperature treatments. In the R subfamily, *MaVAMP72c* expressed the highest expression in both high and low temperature treated leaves, followed by *MaVAMP72a* and *MaYKT6a*; the expression of *MaVAMP72a* and *MaVAMP72b* in low temperature treated banana leaf increased to 5.62- and 4.12-fold of CK, respectively; the expression of *MaYKT6a* and *MaVAMP72b* in high temperature treated banana leaf was, respectively, up-regulated to 7.05- and 3.35-fold of CK, while the expression of *MaTYN12* was down-regulated to only about 16.1% of CK.

#### 2.7.3. *S. indica* and FocTR4 Treatments Influenced the Expression of MaSNAREs in Banana Root

**Figure 7:**
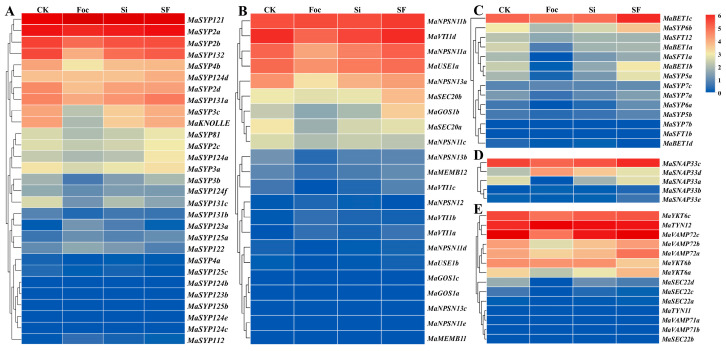
Heatmap for the expression of *MaSNAREs* in *Foc*TR4-, *S. indica*-, and their co-treated banana roots. (**A**–**E**) Qa, Qb, Qc, Qbc and R subfamily, respectively. CK: banana roots without *S. indica* and *Foc*TR4 treatments. Average FPKM values of three biological replications were used for the heatmap drawing. The redder the color, the higher the expression level; the bluer, the lower the expression level.

The expression changes of *MaSNAREs* in banana roots in response to the mutualistic fungus *S. indica* colonization (Si group), pathogenic pathogen *Foc*TR4 infection (Foc group) and their co-treatment (SF group, *S. indica*-colonized seedlings were subjected to *Foc*TR4 infection) were studied (Figure 7). In roots of the Foc group, the expression of *MaSYP121*, *MaSYP112*, *MaSYP122*, *MaSYP124c*, *MaSYP124e* and *MaSYP125a* from the Qa subfamily was up-regulated, and *MaSYP121* was the most significantly up-regulated one, which was about 2.44-fold of CK. The expression levels of *MaKNOLLE* and *MaSYP132* decreased significantly, only accounting for 17.34% and 22.33% of CK, respectively. In the Si group, the expression levels of the six *Foc*TR4 inducible Qa subfamily members were also up-regulated. Compared with the Foc group, however, the expression level of *MaSYP121* in SF group decreased, accounting for about 62.65% of that in Foc group.

The expression of 14 Qb subfamily members were down-regulated by *Foc*TR4, and the expression of *MaNPSN13a* in the Foc group was only 40.96% of CK. The expression of two Qb members, *MaVTI1b* and *MaNPSN12*, was up-regulated by *Foc*TR4 for 8.92- and 5.25-fold, respectively. Compared with CK, the expression trend of Qb subfamily members in Si group was basically similar to that of the Foc group. Compared with Foc group, the expression levels of *MaGOS1a* and *MaVTI1d* in SF group were up-regulated to 4.71- and 2.06-fold of Foc group, respectively.

In the Qc subfamily, the expression levels of *MaSYP7a* and *MaSYP7c* increased after *Foc*TR4 treatment, while the expression levels of the other 11 subfamily members decreased. Compared with CK, the expression level of *MaSYP7c* in the Si group slightly increased, while the other members decreased. Compared with the Foc group, except *MaBET1a* and *MaSYP6b*, the expression levels of other Qc subfamily members all increased in the SF group. It is worth noting that *MaSFT12* and *MaSYP7b* showed very low expression in the Foc group, but their expression levels were much higher in the SF group than in the CK group.

In the Qbc subfamily, the expression of *MaSNAP33b* in the Foc group was about 4.8-fold of CK. While, the expression of *MaSNAP33a* was down-regulated to only 3% of the CK. After *S. indica* colonization, the expression level of *MaSNAP33a*, *MaSNAP33b* and *MaSNAP33d* increased to 4.17-, 3.29- and 2.9-fold of CK, respectively, while the expression levels of *MaSNAP33a* decreased to about 50% of CK. Compared with the Foc group, *MaSNAP33a* and *MaSNAP33c* were up-regulated and *MaSNAP33b* and *MaSNAP33e* were down-regulated in the SF group.

In the R subfamily, the expression of 11 members was down-regulated by *Foc*TR4, and the expression of *MaVAMP71b* and *MaVAMP72a* in the Foc group were only about 31.77% and 22.33% of the CK group, respectively. After *S. indica* colonization, the expression level of *MaTYN12* increased, while the other members decreased. Compared with the Foc group, except for *MaVAMP72c* and *MaYKT6c*, all the other R family *MaSNARE* members were up-regulated in the SF group, and the expression of *MaVAMP72a* was significantly up-regulated to 4.58-fold of the Foc group.

**Figure 8:**
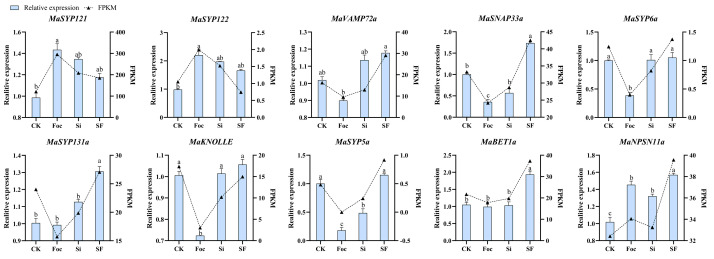
qRT-PCR analysis results of 10 selected *MaSNARE*s. Different lowercase letters above the columns represent significant differences at *p* < 0.05 level.

The expression changes of 10 selected *MaSNAREs* (*MaSYP121*, *MaSYP122*, *MaVAMP72a*, *MaSNAP33a*, *MaSYP6a*, *MaKNOLLE*, *MaSYP5a* and *MaBET1a*, *MaSYP131a* and *MaNPSN11a*) that showed high expression levels in roots and exhibited expression changes in response to *S. indica* or *Foc*TR4 treatments were further validated by using quantitative real time PCR (qRT-PCR) (Figure 8). Results showed that their changing trend was basically consistent with our transcriptome data. Compared with CK, the expression level of *MaSYP121*, *MaSYP122* and *MaNPSN11a* after *Foc*TR4 treatment significantly increased, which was 2.23-, 1.45- and 1.43-fold of CK group, respectively. The expression levels of *MaSYP6a*, *MaKNOLLE*, *MaSYP5a* and *MaSNAP33a* were significantly down-regulated by *Foc*TR4, which account for 38.78%, 71.91%, 18% and 35.79% of CK, respectively. After *S. indica* colonization, the expression levels of eight selected genes (except *MaSYP5a* and *MaSNAP33a*) were up-regulated, and the expression of *MaNPSN11a* increased to about 1.3-fold of CK. Compared with Foc group, the expression levels of *MaVAMP72a*, *MaSYP131a*, *MaSYP6a*, *MaKNOLLE*, *MaSYP5a*, *MaBET1a*, *MaSNAP33a* and *MaNPSN11a* in SF group were significantly higher, accounting for 1.31-, 1.32-, 2.71-, 1.46-, 6.4-, 1.95-, 1.08- and 4.83-fold of the Foc group, respectively.

### 2.8. Foc Resistance Assays in Tobacco Leaves Transiently Overexpressing MaSNAREs

**Figure 9:**
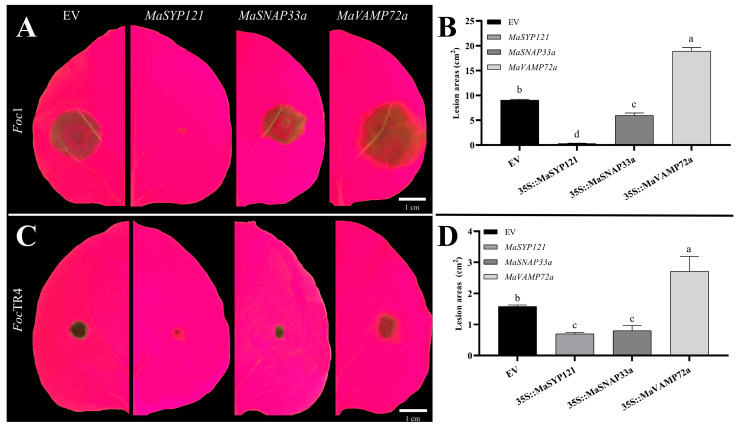
The influence of *MaSYP121*, *MaSNAP33a* and *MaVAMP72a* transient overexpression on *Foc*1 and *Foc*TR4 infection in *N. benthamiana* leaves. (**A**,**B**) lesions in the *N. benthamiana* leaves inoculated with *Foc*1 (at 48 h post inoculation) and *Foc*TR4 (at 72 h post inoculation). (**C**,**D**) lesion areas caused by *Foc*1 and *Foc*TR4 inoculation in the *N. benthamiana* leaves. Different lowercase letters above the columns represent significant differences at *p* < 0.05 level. EV: empty vector control.

The expression levels of *MaSYP121* and *MaVAMP72a* in banana root both ranked top three among all *MaSNAREs*. The Qbc subfamily member *MaSNAP33a* was also highly expressed in roots. Both our transcriptome and qRT-PCR results showed that the expression of *MaSYP121* was up-regulated by *Foc*TR4 and *S. indica*. According to our transcriptome data, the expression of *MaVAMP72a* and *MaSNAP33a* was up-regulated by both fungi. Moreover, their *Foc*TR4 responsive characteristic was greatly alleviated in the SF group. Therefore, these three members were predicted to play important roles in the *S. indica*–banana–*Foc* interactions. To verify their functions, overexpression vectors for *MaSYP121*, *MaVAMP72a* and *MaSNAP33a* were constructed, transiently overexpressed in tobacco leaves and subjected to *Foc* Race 1 (*Foc*1) and *Foc*TR4 inoculation (Figure 9). After *Foc*1 and *Foc*TR4 inoculation, the lesion areas in the tobacco leaves expressing *MaSYP121* were significantly smaller than the empty vector controls (EV), accounting for only 3.19% and 44.18% of EV, respectively. Transient overexpression of *MaSNAP33a* also reduced the lesion area, and the *Foc*1- and *Foc*TR4-caused lesion area in tobacco leaves overexpressing *MaSNAP33a* was only 65.67% and 50.66% of EV, respectively. However, transient overexpression of *MaVAMP72a* resulted in larger *Foc*1- and *Foc*TR4-caused lesion areas in tobacco leaves, which was 2.07- and 1.72-fold of EV, respectively.

## 3. Discussion

### 3.1. Segmental Duplications Contributed to the Expansion of Banana SNARE Gene Family

The intracellular transportation in eukaryotic cells is largely dependent on vesicle trafficking [3]. As a key participant of vesicle trafficking, SNAREs are crucial for the membrane fusion between vesicles and target membranes [8]. The large number of *SNAREs* in plant genomes were reported to be closely related to the increased multicellular nature of plants [22] and their miscellaneous functions [40]. Plant SNAREs can be divided into 5 subfamilies (Qa-, Qb-, Qc-, Qbc- and R-SNARE) and 24 groups [41,42,43]. Consistently, in this study, we obtained the same classification results for MaSNAREs. The amount of *MaSNARE* members is approximately 1.31 times that of Arabidopsis and 1.4 times that of rice, which may be related to the multicellular and triploid nature of banana [44]. We identified 23 segmental duplicated gene pairs involving 34 *MaSNAREs* from banana. In tomato, segmental duplications were also reported to be main contributors for the expansion of the *SNARE* gene family [21]. Moreover, among these segmental duplicated tomato SNARE genes, members from VAMP groups were the most abundant, followed by SYP1 members. Consistently, in this study, *MaSNARE* members from the VAMP group (five members) and SYP1 group (five members) also ranked top two.

### 3.2. The Expression of MaSNAREs Varied a Lot in Different Organs

*SNAREs* are expressed in various tissues of plants and participate in almost all plant growth and development processes. Evidence has shown that their expression patterns and functions in different tissues and organs varied [6]. For example, *SYP132* is expressed in all tissues and organs of Arabidopsis, while *SYP124*, *SYP125* and *SYP131* are only expressed in pollen, and *SYP123* is only expressed in root hair cells [26]. In this study, we found that there were 81, 69, 58 and 9 *MaSNAREs* that showed expression in banana leaf, root, corm and fruit, respectively. In *Vaccinium myrtillus*, *SYP5*, *SYP6*, *SYP7*, *SEC20*, *SEC22* and *YKT6* genes were expressed in fruit [45]. Our study found that, among the nine fruit-expressing *MaSNAREs*, *MaSYP6b* (homologous to *V. myrtillus SYP6*) showed the highest expression level, and *MaSEC22a* (homologous to *V. myrtillus SEC22*) also showed high expression in fruit, suggesting that they might function a lot in banana fruit.

### 3.3. The Expression of MaSNAREs Could Be Significantly Influenced by Many Phytohormones and Environmental Factors

Evidence has revealed that phytohormones influenced greatly the expression and functions of *SNARE* genes [46,47,48]. The expression of Arabidopsis *SYP132* could be significantly affected by auxin [46]. In this study, 52.38% of the *MaSNAREs* contained auxin response elements in their promoters. *NtSYR1* (a homologous gene of *AtSYP121* in tobacco) was proved to function in regulating the growth and development of plants by affecting the ABA response process [47]. Consistently, our study revealed that 77.38% of *MaSNARE* promoters contained the ABA responsive elements. SNARE proteins play important roles in plant responses to both abiotic and biotic stresses [6]. Their regulatory roles in Ca^2+^ signaling pathway and ABA signaling have been proved to be vital for enhancing the salt, alkali and drought resistance of plants [28]. In consistence with this, we found that 94.05% of *MaSNAREs* contained drought inducibility elements in their promoters. *AtSYP121* and *AtSYP122* have been proved to be negative regulators of SA, JA and ET pathways [48]. In this study, we found that 78.57% of *MaSNARE* promoters had MeJA responsive *cis*-acting elements and many SA and ET responsive elements were also identified in their promoters.

Additionally, 90.48% and 48.81% of *MaSNAREs* contained high-temperature and low-temperature responsive elements in their promoters, respectively. Gene expression analysis showed that many *MaSNAREs* were temperature stress responsive. For example, *MaVAMP72a*, *MaNPSN11a*, *MaSYP121* and *MaBET1d* were up-regulated by low temperature more than twofold. All of them, except *MaSYP121*, had low-temperature responsive element LTR. The MaSYP121 promoter contained the most SA-responsive elements (accounting to eight), which might explain why it can be so significantly up-regulated by low temperature. Moreover, four high-temperature-inducible *MaSNAREs*, including *MaYKT6a*, *MaSYP81*, *MaSYP131c* and *MaSYP2d*, all contained high-temperature responsive element STRE in their promoters. This suggested that the presence of these elements function accordingly as in other plants, resulting in a correlation in the *MaSNAREs*’ expression during the encountered temperature stress conditions.

### 3.4. MaSYP121 and MaSNAP33a Function in S. indica-Banana-Foc Interactions

The SNARE protein complex (composed of AtVAMP721/722, AtSYP121, AtSYP122 and AtSNAP33) is necessary for Arabidopsis to resist powdery mildew [39]. In this study, *MaSYP121*, *MaVAMP72a* and *MaSNAP33a* were identified to be homologous genes of Arabidopsis *SYP121* (*PEN1*), *VAMP721* and *SNAP33* (Supplemental Table S1), respectively. The expression levels of Arabidopsis *PEN1* could be significantly up-regulated by *E. cichoracearum* inoculation, and PEN1 appeared to be actively recruited to the papillae during fungal attack, thereby enhancing powdery mildew resistance [49]. In rice, the expression of *OsSYP121* could be significantly up-regulated after rice blast fungus *Magnaporthe griseat* inoculation, and transgenic plants overexpressing *OsSYP121* exhibited enhanced resistance to rice blast [50]. Our study revealed that the expression of *MaSYP121* was induced by *Foc*TR4, suggesting that it functions in the banana in response to Fusarium wilt. Notably, *S. indica* treatment alleviated the up-regulation of *MaSYP121* by *Foc*TR4, which might be related to the enhanced disease resistance conferred by *S. indica*. Through pathogen resistance assays in tobacco leaves, we found that its overexpression greatly suppressed the infection of both *Foc*1 and *Foc*TR4, indicating that this gene functioned positively in resisting *Foc* penetration.

The SNARE protein complex formed by barley HvSNAP34 (homologous protein of AtSNAP33) and HvSYP121 (homologous protein of AtSYP121) is crucial in the process of resistance to barley powdery mildew [26]. Our study revealed that *MaSNAP33a* was down-regulated while *MaSNAP33b* and *MaSNAP33e* were up-regulated by *Foc*TR4, by *Foc*TR4. However, *S. indica* up-regulated greatly the expression of *MaSNAP33a*, *MaSNAP33b* and *MaSNAP33e*. *Foc* resistance assays in tobacco leaves revealed that, although its inhibitory effect is not strong as that of *MaSYP121*, the overexpression of *MaSNAP33a* could also suppress the infection of both *Foc*1 and *Foc*TR4.

The expression of *MaVAMP72a* was down-regulated by *Foc*TR4, however, its expression level was significantly up-regulated by *S. indica* and Si-*Foc*TR4 treatment. Unlike *MaSYP121* and *MaSNAP33a*, the overexpression of *MaVAMP72a* improved the growth and infection in both *Foc*1 and *Foc*TR4. The differential expression changes the patterns of these three *MaSNAREs* under mutualistic and pathogenic fungal colonization and their diverse effects in resisting *Foc* infection suggested that they have different functions. Their functional mechanisms need to be further explored in the future research. However, it can be concluded that *MaSYP121* and *MaSNAP33a* function positively in resisting *Foc* infection.

## 4. Materials and Methods

### 4.1. Fungal Strains and Plant Materials Used in This Study

The *Serendipita indica*, *Foc*TR4 and *Foc*1 strains used in this study were provided by the Institute of Horticultural Plant Biotechnology, Fujian Agriculture and Forestry University. The 30-day-old tobacco (*N. benthamiana*) seedlings used for transient overexpression were provided by Shanxi Agricultural University and cultured in an artificial climate culture chamber maintained at 28 °C, 80% relative humidity, and a photoperiod of 16 h light (1500 ± 200 lx)/8 h dark.

### 4.2. Identification and Bioinformatic Analysis of Banana SNARE Genes

The banana (*Musa acuminata*) gDNA, CDS and protein sequences and genome annotation files were downloaded from the banana genome website (https://banana-genome-hub.southgreen.fr/, accessed on 4 July 2022). A total of 64 Arabidopsis SNARE family protein sequences were downloaded from TAIR (https://www.arabidopsis.org, accessed on 4 July 2022) and used as query sequences to BLASTP against the banana protein database using E ≤ 1 × e^−5^ as criterion. Following this, the hidden Markov model files for SNARE (PF05739), Syntaxin (PF00804), Longin (PF13774), Synaptobrevin (PF00957), SEC20 (PF03908), V-SNARE-C (PF12352), V-SNARE (PF05008) and USE1 (PF09753) were downloaded from the Pfam database (http://pfam.xfam.org/, accessed on 4 July 2022) and searched against the banana genome data using HMMER software (E-value ≤ 1 × 10^−5^). The identified candidate proteins were further subjected to domain validation by using CDD (http://smart.embl-heidelberg.de/, accessed on 4 July 2022), and sequences with none of the conserved domains were removed. The physicochemical properties, signal peptides, transmembrane structure and subcellular localization of remained MaSNAREs were predicted using ExPASy (https://web.expasy.org/protparam/, accessed on 5 July 2022), SignalP 3.0 Server (http://www.cbs.dtu.dk/services/SignalP-3.0/, accessed on 5 July 2022), TMHMM Server v.2.0 (http://www.cbs.dtu.dk/services/TMHMM/, accessed on 5 July 2022) and Euk-mPLoc 2.0 (http://www.csbio.sjtu.edu.cn/bioinf/euk-multi-2/, accessed on 5 July 2022), respectively.

The conserved motifs of MaSNAREs were analyzed using the MEME (http://meme-suite.org/tools/meme, accessed on 6 July 2022) with the default value set as 20. GSDS (http://gsds.cbi.pku.edu.cn/, accessed on 6 July 2022) was used for gene structure analysis of *MaSNAREs*. The generated conserved motifs and gene structure results were visualized using TBtools [51].

### 4.3. Phylogenetic Analysis of Banana SNARE Genes

Multiple sequence alignment of SNARE proteins from banana and *A. thaliana* was performed using the MUSCLE method, and the phylogenetic tree was constructed using the maximum likelihood method of MEGA-X software (Jones-Taylor-Thornton (JTT) model, complete deletion and bootstrap values were 1000 replicates).

### 4.4. Chromosome Location and Gene Duplication Analysis of Banana SNAREs

According to the banana genome annotation information, the chromosome locations of *MaSNAREs* were displayed using TBtools [51]. MCscanX software was used to analyze the gene duplication events among *MaSNAREs*. By using Ka/Ks_Calclator 2.0 software, the synonymous substitution value (*Ka*) and synonymous substitution value (*Ks*) of the identified duplicated *MaSNARE* genes were calculated [52]. The divergence time (T) of gene duplication event was calculated using the formula: T = Ks/2λ × 10^−6^ Mya (λ: synonymous substitution rate; λ = 4.5 × 10^−9^) [53].

### 4.5. Prediction of Cis-Acting Elements in MaSNAREs Promoters

The 2000 bp sequences upstream of the initiation codon (ATG) of *MaSNAREs* were extracted from the banana genome data using TBtools and used as promoter sequences. PlantCARE (http://bioinformatics.psb.ugent.be/webtools/plantcare/html/, accessed on 6 July 2022) was used to predict *cis*-acting elements in the *MaSNAREs* promoters.

### 4.6. Expression Analysis of MaSNAREs

The FPKM values of *MaSNAREs* in root, corm, leaf and fruit, in leaves treated at 4 °C for 24 h (low temperature treatment), 45 °C for 3 d (high temperature treatment) and 28 °C (control), and in roots treated by *Foc*TR4 (Foc group, harvested at two months post *Foc*TR4 inoculation), *S. indica* (Si group), *S. indica* and *Foc*TR4 co-treated (SF group, harvested at two months post *Foc*TR4 inoculation) and non-inoculated control group (CK group) were extracted from the transcriptome data. Following this, their expression values were normalized by log_2_ (FPKM + 1) and used for the heatmap drawing using Heatmap embedded in TBtools.

RNAprep Pure Plant Kit (TIANGEN, Beijing, China) was used to isolate RNA from CK, Foc, Si and SF root samples, and cDNA was synthesized using PrimerScript^TM^ RT Reagent Kit (Perfect Real Time) (TaKaRa, Dalian, China). By using Primer3 (https://primer3.ut.ee/, accessed on 10 July 2022), primers used for the quantitative real time PCR analysis of 10 selected genes (including *MaSYP121*, *MaSYP122*, *MaVAMP72a*, *MaSNAP33a*, *MaSYP6a*, *MaKNOLLE*, *MaSYP5a*, *MaBET1a*, *MaSYP131a* and *MaNPSN11a*) were designed (Table S4). A qRT-PCR analysis was performed for the validation of the expression change patterns of the 10 selected *MaSNARE* genes in the roots of the F, Si, SF and CK groups. The qRT-PCR reactions were performed on Roche Light-Cycler480 with the *CAC* as the internal reference gene. The 25 μL qRT-PCR system contained 12.5 μL of Dream Taq^TM^ Green PCR Master (2×), 9.5 μL of ddH_2_O, 1 μL of template cDNA, and 1 μL each of the upstream and downstream primers. The reaction conditions were as follows: pre-denaturation at 95 °C for 3 min, denaturation at 95 °C for 5 s, annealing at 60 °C for 30 s, extension at 72 °C 15 s, 40 cycles. The relative expression of *MaSNAREs* genes in different groups was calculated using the 2^−ΔΔCT^ method. SPSS 26.0 software was used for significance analysis and GraphPad Prism 8 software was applied for figure drawing.

### 4.7. Gene Cloning and Vector Construction

DNAMAN was used to design gene-specific primers for cloning the full-length coding sequences (CDSs) of *MaSYP121*, *MaVAMP72a* and *MaSNAP33a* (Table S4). The cDNA was synthesized using the RevertAid First-strand cDNA synthesis Kit (Thermo Scientific, Shanghai, China). The 25 μL gene amplification system contained 1 μL cDNA, 1 μL each of forward and reverse primers, 12.5 μL 2 × Green mix and 9.5 μL ddH_2_O. The amplification procedure was as follows: pre-denaturation at 95 °C for 3 min, denaturation at 95 °C for 20 s, annealing at 57 °C for 30 s, extension at 72 °C for 1 min, 35 cycles; and final extension at 72 °C for 10 min. The PCR product was gel-extracted, ligated into 18-T vector and transformed into *E. coli* DH5α. Positive clones were selected and sent to Sangon Biotech (Shanghai) Co., Ltd. for sequencing verification. According to the sequencing results, primers for *MaSYP121*, *MaVAMP72a* and *MaSNAP33a* vector constructions were further designed. By using the TA plasmid-carrying target gene as a template, target genes with the same homologous arm sequences were amplified, gel-extracted, ligated into the pBI123 vector using a ready-to-use seamless cloning kit (Sangon Biotech, Shanghai, China) and transformed into *Agrobacterium* GV3101 for further use.

### 4.8. Foc Resistance Assays in Tobacco Leaves Transiently Overexpressing MaSYP121, MaVAMP72a and MaSNAP33a

*Agrobacterium* GV3101 carrying pBI121-MaSYP121, pBI121-MaVAMP72a and pBI121-MaSNAP33a recombinant plasmids and empty vector (EV, as control) were shake-cultured to OD_600_ = 1.5–2.0, centrifugated at 6000 rpm for 10 min to remove supernatant solutions, re-suspended with buffer (containing 10 mM/L MgCl_2_ + 10 mM/L MES + 100 μM/L acetosyringone (As), pH = 5.8), adjusted to OD_600_ = 0.8–1.0, and activated by shaking at 28 °C at 200 rpm for 20–30 min [54]. Leaves from 30-day-old *N. benthamiana* plants were gently pricked with a needle and injected with a volume of about 40 μL *Agrobacterium* solution using a 1 mL syringe from the abaxial side. Following this, the tobacco plants were removed to a culture chamber (25 ± 2 °C, relative humidity ~80%) for dark culture. Two days later, leaves were harvested and used for *Foc* inoculation.

*Foc*1 and *Foc*TR4 fungi that had been cultured on PDA plates for 7 days were used as inoculation materials. The pathogen discs with a diameter of 5 mm were made and placed on the back of the tobacco leaves. The leaves were then placed on a moist filter paper in a petri dish with a diameter of 90 mm. To maintain a high humidity environment, leaf petioles were wrapped with moist cotton. The culture dishes were placed in a constant temperature incubator (28 ± 1°C, 12 h light/12 h dark photoperiod). After 48 h, leaf lesions in *Foc*1 inoculated tobacco leaves were photographed with UV imaging and the lesion area was calculated. As the lesions caused by *Foc*TR4 were much smaller than that caused by *Foc*1, leaf lesions in *Foc*TR4 inoculated tobacco leaves were observed at 72 h post inoculation. The lesion areas in *MaSYP121*, *MaVAMP72a* and *MaSNAP33a* overexpressing leaves were compared with leaves overexpressing the EV to show their inhibitory effect [55]. This experiment was performed with three biological replications, of which each was a mixed sample of six leaves.

## 5. Conclusions

In this study, 84 *SNARE* genes which can be further divided into Qa-, Qb-, Qc-, Qbc- and R-SNARE subfamilies were identified from the *M. acuminata* genome. The gene duplication events analysis revealed that segmental duplications contributed greatly to the expansion of this gene family. A large number of phytohormones and stress-responsive elements were identified from *MaSNAREs* promoters, suggesting that their expression could be influenced by phytohormones and many other factors. Consistently, the expression of some *MaSNAREs* containing low-temperature and high-temperature responsive elements in their promoters were influenced by high and low temperature stresses. In addition, we found that the expression of many *MaSNAREs* was affected by *Foc*TR4 and *S. indica* infection. Pathogen resistance assays in tobacco leaves showed that *MaSYP121* and *MaSNAP33a* overexpression could inhibit the penetration of both *Foc*1 and *Foc*TR4, suggesting that they play positive roles in resisting *Foc* infection in banana. The functions of *MaSNAREs* in banana stress responses should be focused on in future studies. Our study will be helpful for understanding the roles of MaSNAREs in banana and can provide valuable gene resources for banana resistance breeding in the future.

## Data Availability

The data supporting reported results can be found in the manuscript and supplemental materials.

## Supplementary Materials

The following supporting information can be downloaded at: https://www.mdpi.com/article/10.3390/plants12081599/s1, Table S1: Basic physicochemical properties of banana SNARE proteins; Table S2: *Cis*-acting elements with unknown functions in promoters of banana *SANRE*s; Table S3: The calculated *Ka*/*Ks* values for the segmental duplicated gene pairs in banana *SNARE* gene family; Table S4: Information of primers used in this study. Figure S1: Phylogenetic trees constructed using 84 MaSNAREs and 64 AtSNAREs.
